# Case of Protracted Gestation

**Published:** 1859-05

**Authors:** W. R. Stone

**Affiliations:** Manhattan, Indiana


ARTICLE V.

### CASE OF PROTRACTED GESTATION.

BY DR. W. R. STONE, OF MANHATTAN, INDIANA.

*Case.*—Mrs. K., aged twenty-four years, sanguine-bilious temperament, and of usually good health, lost her husband on the 17th of March, 1858, from which time, as the catamenia did not manifest itself, she deemed herself probably pregnant, which supposition induced her to consult me as early as the last of May, 1858, when I gained from her the following history:

Last menstruation on the 25th of February, 1858. Last sexual intercourse on the 10th of March, 1858. Feels somewhat different from a former gestation (having had one previous gestation), especially in an absence of gastric irritation, with which she was much troubled in the first.

As no urgent symptoms were present, I dismissed my patient, by informing her that she was likely pregnant, and by requesting her to acquaint me with any derangement of her general health that might occur, when we would more fully investigate her condition.

No further information of the case till the 13th of July, 1858, when she informed me that undoubted quickening had occurred on the 8th of July, making one hundred and thirty-three days from last catamenia, and one hundred and twenty days from last sexual intercourse.

From this time up to the 15th of December, nothing of unusual interest transpired, at which time (15th December), "spurious pains" occurred, lasting twelve hours, and regularly occurring on the eighth day, for five weeks, when they came up every second or third day, till the 3d of February, 1859, at which time, and with a favorable labor, she was delivered of a female child, weighing eight lbs.

During the last two months of gestation, the movements of the fœtus were very strong, causing much suffering to the mother.

*Resume of dates:*

| | | | | |
|---|---|---|---|---|
| Number of days from last catamenia to delivery, | | | | 343 |
| " | " | " | sex. inter. " | 330 |
| " | " | " | catamen. to quickening, | 133 |
| " | " | " | sexual cong. " | 120 |

*Development of Infant.*—The osseous system was extraordinarily well developed, the sub-cutaneous processes being well fitted, and firm; the articulations firm and compact; the cranial bones immovably united by the sutures, and the posterior fontanelle entirely closed,—the anterior also closed by the inner tablet, inasmuch as no pulsation was observable. The skin was freely organized, and of that fine tint characteristic of a three months infant. In a word, the general appearance of this infant was fully up to that of most infants three months old.

*Peculiarities of Placenta, Liquor Amnii, etc.*—About one-fourth of the structure of the placenta was osseous matter, arranged in lamella, and irregular granules. The placenta about the usual size. The liquor amnii normal in amount, of a light straw color, and quite viscid; uniting, also, a strong, urinous odor. The membranes of sufficient firmness to form a basket that would support the child's weight, no doubt.

*Character of Mrs. K.*—The lady has always sustained an irreproachable deportment, and stood high in the estimation of those knowing her intimately. Indeed, she is distinctly reserved, and modest in her social intercourse with friends.

*Opinion and Character of the Physicians who have seen, and are acquainted with the circumstances of the case.*—The following physicians have seen, and examined the infant: Drs. Layman, Knight, Heavenridge, W. M. Denny, and R. B. Denny. They all entertain the opinion that this was a case of protracted gestation, and are fully satisfied that the child is legitimate.

It is but just to say of the above named physicians, that they sustain a good character with their professional brethren, and deservedly, too, as they are qualified for their business.

With this brief history, the case is submitted to the profession, only adding the assurance, that no effort has been made to "bolster up" the case on false statements, but a plain statement of facts have been aimed at.

